# The Role of Tetrahydrobiopterin and Dihydrobiopterin in Ischemia/Reperfusion Injury When Given at Reperfusion

**DOI:** 10.1155/2010/963914

**Published:** 2010-06-09

**Authors:** Qian Chen, Elizabeth Eun Jung Kim, Katrina Elio, Christopher Zambrano, Samuel Krass, Jane Chun-wen Teng, Helen Kay, Kerry-Anne Perkins, Sailesh Pershad, Sloane McGraw, Jeffrey Emrich, Jovan S. Adams, Lindon H. Young

**Affiliations:** Department of Pathology, Microbiology and Immunology & Forensic Medicine, Philadelphia College of Osteopathic Medicine, 4170 City Avenue, Philadelphia, PA 19131-1694, USA

## Abstract

Reduced nitric oxide (NO) bioavailability and increased oxidative stress are major factors mediating ischemia/reperfusion (I/R) injury. Tetrahydrobiopterin (BH_4_) is an essential cofactor of endothelial NO synthase (eNOS) to produce NO, whereas dihydrobiopterin (BH_2_) can shift the eNOS product profile from NO to superoxide, which is further converted to hydrogen peroxide (H_2_O_2_) and cause I/R injury. The effects of BH_4_ and BH_2_ on oxidative stress and postreperfused cardiac functions were examined in ex vivo myocardial and in vivo femoral I (20 min)/R (45 min) models. In femoral I/R, BH_4_ increased NO and decreased H_2_O_2_ releases relative to saline control, and these effects correlated with improved postreperfused cardiac function. By contrast, BH_2_ decreased NO release relative to the saline control, but increased H_2_O_2_ release similar to the saline control, and these effects correlated with compromised postreperfused cardiac function. In conclusion, these results suggest that promoting eNOS coupling to produce NO and decrease H_2_O_2_ may be a key mechanism to restore postreperfused organ function during early reperfusion.

## 1. Introduction

The further damage caused by reperfusion following ischemia (I/R) has been a crucial event to attenuate in order to preserve more tissue or organ function [1, 2]. Understanding mechanisms related to I/R injury in clinical cases involving myocardial infarction, coronary bypass/angioplasty, and organ transplantation provides a basis to develop new targets of intervention to attenuate the pathophysiological process. Reduced nitric oxide (NO) bioavailability and increased oxidative stress are major factors mediating I/R injury [3]. It has been shown that endothelium-derived NO is abruptly decreased within 5 minutes of reperfusion that results in endothelial dysfunction in myocardial I/R (MI/R) injury [3]. Endothelial dysfunction serves as a trigger to initiate the upregulation of endothelial cellular adhesion molecules to promote polymorphonuclear leukocyte (PMN) adherence and infiltration [3–5]. Subsequently, the transmigrated PMNs release cytotoxic substances such as superoxide (SO) radicals to directly injure the myocardium and cause cardiac contractile dysfunction [4, 6]. 

It is well known that vascular NO is produced by endothelial NO synthase (eNOS) by converting L-arginine to L-citrulline in the presence of molecular oxygen. Tetrahydrobiopterin (BH_4_) is an essential cofactor of eNOS to aid in the coupling between L-arginine and the heme site within the oxygenase domain of eNOS [7]. Under this coupled state, eNOS produces NO to facilitate normal blood flow and to maintain an antiinflammatory and antithrombotic vascular endothelial surface [8]. By contrast, dihydrobiopterin (BH_2_), an oxidized form of BH_4_, can cause uncoupling between L-arginine and eNOS. Under this condition, eNOS utilizes molecular oxygen as the substrate in the absence of L-arginine to generate SO instead of NO [9–11]. SO is further converted into hydrogen peroxide (H_2_O_2_) by superoxide dismutase. Moreover, incomplete oxidative phosphorylation in dysfunctional mitochondria in murine endothelial cells can facilitate additional oxidation of BH_4_ to BH_2_, thus increasing the BH_2_ to BH_4_ ratio to further promote eNOS uncoupling [12, 13]. With this regard, eNOS uncoupling may induce NO insufficiency, but also contribute to the oxidative stress under various pathological conditions, such as I/R. Previous studies evaluated that increasing eNOS activity with PKC epsilon activator resulted in compromised postreperfused heart function, whereas decreasing eNOS activity with PKC epsilon inhibitor resulted in restoration of postreperfused cardiac function associated with decreased H_2_O_2_ release in rat femoral artery/vein subjected to I/R [12]. By contrast, a nonselective NOS inhibitor, *N* ^*G*^-nitro-L-arginine methyl ester (L-NAME), does not appreciably inhibit SO release from NOS and consequently is not associated with improvement of cardiac function in many MI/R models [11, 14]. 

Regarding BH_4_, previous studies have shown that BH_4_ given prior to myocardial ischemia significantly restored post-reperfusion cardiac function in the isolated perfused rat heart and enhanced nitrite levels in the coronary perfusate [15]. Although, the potential opposing effects of BH_2_ were not investigated in the Yamashiro et al. study. In addition, the role of BH_4_ or BH_2_ mediating eNOS coupling or uncoupling exclusively during the early reperfusion has not been assessed in real-time in vivo or ex vivo. This is clinically important in that pretreatment may not be an option in clinical settings whereas treatment during the early reperfusion would be more practical and helpful to improve the organ function.

 Therefore, in this study, BH_4_ was used during the early reperfusion within 5 minutes to promote the eNOS coupled state, whereas BH_2_ was utilized to further maintain the eNOS uncoupled state. The following hypotheses were tested. Firstly, the effects of BH_4_ or BH_2_ on vascular NO release were tested by directly measuring NO release from nonischemic rat aortic segments and from femoral arteries/veins subjected to I/R in anesthetized rats. Secondly, the effects of BH_4_ or BH_2_ on oxidative stress (i.e., H_2_O_2_) were recorded by directly measuring H_2_O_2_ release from femoral arteries/veins subjected to I/R in anesthetized rats. Thirdly, the effects of BH_4_ or BH_2_ on postreperfused cardiac function and PMN vascular adherence/infiltration were determined in a PMN-induced MI/R injury animal model. Then L-NAME was used to test if the cardioprotection and decreased PMN adherence/infiltration provided by BH_4_ could be blocked to indicate a NO mechanism involved in this cardioprotection. 

## 2. Methods

### 2.1. In Vitro Measurement of NO Release from Rat Aortic Segments

The Institutional Animal Care and Use Committee of Philadelphia College of Osteopathic Medicine approved all animal protocols performed in this study. As previously described, the isolated rat aortas were cut to 6-7 mm long (i.e., 10 mg wet weight) after removal of adherent fat and connective tissue. Then these aortic rings were cut open and fixed by pins with the endothelial surface facing up in 24-well culture dishes containing 1 mL oxygenated Krebs'-Henselit (K-H) solution maintained at 37°C (in mmol/L: 10.0 dextrose, 119.0 NaCl, 12.5 NaHCO_3_, 2.5 CaCl_2_, 4.8 KCl, 1.2 KH_2_PO_4_, and 1.2 MgSO_4_). The NO release was measured using a calibrated NO meter (Iso-NO; World Precision Instruments, Sarasota, FL) connected to a polygraph internally shielded NO electrode [16]. Basal rat aortic endothelial NO release was determined by the difference of reading between a well containing only K-H buffer and the well containing aortic tissue as in previous studies [17]. After basal NO measurement, the effects of acetylcholine (Ach, 5 *μ*M) and BH_4_ (1–40 *μ*M) or BH_2_ (50–200 *μ*M) were determined. Ach was used as a positive control to assess the viability of the endothelium. Then 800 *μ*M L-NAME was added to the K-H buffer solution; the effects of Ach (5 *μ*M), BH_4_ (10 *μ*M), or BH_2_ (100 *μ*M) were reassessed 30 minutes later to evaluate if L-NAME could block NO release. All measurements were reported in picomoles per milligram of aortic tissue. The number of trials for each group was indicated in Table 1.

### 2.2. In Vivo Measurement of *NO*/*H*_2_ *O*_2_ Release from Rat Femoral Veins during Reperfusion

NO or H_2_O_2_ release was measured from femoral veins in anesthetized rats, one subjected to I/R and the other is a nonischemic sham control. This rat femoral I/R model was based on the procedure of Kuntscher et al. [18]. The NO or H_2_O_2_ microsensors (100 *μ*m diameter) connected to a free radical analyzer (Apollo 4000; World Precision Instruments, Sarasota, FL) were inserted into a catheter and placed inside each femoral vein as previously published [12]. Ischemia of femoral circulation in one side would be induced by clamping the femoral artery/vein for 20 minutes followed by 45 minutes reperfusion via releasing the clamp, which was similar to the MI/R time course. BH_4_ (6.5 mg/kg, which corresponds to about 250 *μ*M in the blood concentration), BH_2_ (2 or 4 mg/kg, which corresponds to about 100 or 200 *μ*M in the blood concentration), or saline (for nondrug control group) was applied through tail vein injection at the beginning of reperfusion. The total volume of solution was 1 mL saline or drug mixed with saline. NO or H_2_O_2_ release was continuously recorded and collected at 5 minutes intervals during a 15-minute baseline period, 20-minute ischemia and 45-minute reperfusion. The changes in NO or H_2_O_2_ release during reperfusion (in pA) were expressed as relative changes to baseline (initial). Thereafter, the values were converted to the concentration of NO (nM) or H_2_O_2_ (*μ*M) after correction to the preexperimental calibration curve of NO or H_2_O_2_ microsensors.

### 2.3. Isolated Rat Heart Preparation

**Figure 1:**
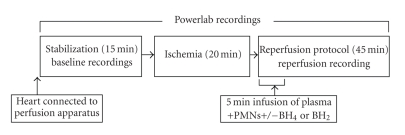
Schematic diagram of the ischemia/reperfusion protocol in the isolated perfused rat heart model.

After injecting pentobarbital sodium (60 mg/kg) and sodium heparin (1,000 U) intraperitoneally (i.p.), hearts were rapidly excised from male Sprague Dawley rats (275–325 g, Ace Animals, Boyertown, PA) [19]. Then the heart was subjected to retrograde perfusion with a modified Krebs' buffer composed of 17.0 dextrose, 120.0 NaCl, 25.0 NaHCO_3_, 2.5 CaCl_2_, 0.5 EDTA, 5.9 KCl, and 1.2 MgCl_2_ (in mmol/L, 37°C, pH of 7.3-7.4). Three side arms in the perfusion line proximal to the heart inflow cannula, allowed perfusing BH_4_ or BH_2_/plasma, PMNs, and L-NAME. Coronary flow was monitored by a flowmeter (T106, Transonic Systems, Inc., Ithaca, NY). Left ventricular-developed pressure (LVDP, defined as left ventricular end-systolic pressure minus left ventricular end-diastolic pressure) and the maximal rate of LVDP (+*dP*/*dt*_max_) were monitored using a pressure transducer (SPR-524, Millar Instruments, Inc., Houston, TX), which was positioned in the left ventricular cavity. LVDP,  +*dP*/*dt*_max_, and coronary flow were measured every 5 minutes for 15 minutes baseline recording; 20-minute global ischemia was induced by stopping perfusion (i.e., turning stop-cock to off position) and 45-minute reperfusion. The schematic protocol is shown in Figure 1. All data were recorded using a Powerlab Station acquisition system (ADInstruments, Grand Junction, CO) in conjunction with a computer (Gateway). 

 The isolated rat heart was cannulated via the aorta onto a perfusion needle and was immersed in a water-jacketed reservoir that contains 160 mL of Krebs' buffer maintained at 37°C. The preload volume came from Krebs' buffer that filled the left ventricle upon insertion of the pressure transducer catheter in the base of the left side of the heart. We also used animals in the same weight range for all study groups and therefore the preload should be similar among all study groups as in previous studies [17, 19, 20]. The initial baseline left ventricular end-diastolic pressure was between 4–8 mmHg for all hearts in each study group. 

### 2.4. Groups of Isolated Perfused Hearts

Table 2 indicates the 12 groups (control and treatment conditions) of isolated perfused rat hearts used in the study. Three types of control groups were used in the study based on the well-established PMN-induced MI/R model [21]. (1) Sham hearts were not subjected to ischemia and are not perfused with PMNs, but they were perfused with 5 mL of plasma (1 mL/min) at 35 minutes into perfusion (i.e., the same time point that I/R hearts would be given 5 mL of plasma: 15 minutes of baseline recordings plus 20 min ischemia). This group was employed to show that cardiac function (i.e., LVDP and +*dP*/*dt*_max_\) could be maintained throughout the 80-minute protocol. (2) I/R hearts were subjected to 20 minutes of ischemia/45 minutes of reperfusion, and were perfused with 5 mL of plasma (1 mL/min) in the absence of PMNs during the first 5 minutes of reperfusion. This group was employed to show that these hearts would recover to near baseline values by the end of 45-minute reperfusion. Twenty-minute ischemia followed by 45-minute reperfusion would stun the heart but was a form of reversible cell injury. (3) I/R+PMN hearts were subjected to 20 minutes of ischemia and were reperfused with PMNs (200 × 10^6^, resuspended in 5 mL Krebs' buffer) and 5 mL of plasma (1 mL/min) during the first 5 minutes of reperfusion. This group was employed to show that 20 minutes of ischemia followed by 45 minutes of reperfusion in the presence of PMNs resulted in a sustained cardiac contractile dysfunction throughout the 45-minute reperfusion period compared to initial baseline values. Previous studies showed that sham hearts given PMNs exhibited no changes from initial control values [19]. This result indicated that PMNs without ischemia were not sufficient to induce sustained cardiac dysfunction. In some sham and I/R hearts, BH_4_ (10 *μ*M) or BH_2_ (100 *μ*M) was dissolved in plasma and infused at a rate of 1 mL/min for 5 minutes at 35 minutes into perfusion (sham) or at the beginning of reperfusion. These groups were employed to show that BH_4_ or BH_2_ did not exert a cardiotonic or cardiodepressant effect in sham or I/R settings at this concentration. In some I/R+PMN hearts, different doses of BH_4_ (1–10 *μ*M) or BH_2_ (100 *μ*M) were infused at a rate of 1 mL/min for 5 minutes at the beginning of reperfusion to test whether BH_4_ or BH_2_ might elicit cardioprotective effects. Furthermore, a NO synthase inhibitor, L-NAME (50 *μ*M), was used throughout the 45 minutes of reperfusion in some BH_4_-treated I/R+PMN hearts to determine if a NO mechanism was involved in the cardioprotective effect. The dose of L-NAME (50 *μ*M) used in this study does not significantly affect LVDP in sham hearts [22].

### 2.5. Isolation of Plasma

Blood was collected from the aorta in citrate phosphate buffer (Sigma Chemical Co., St. Louis, MO) just before isolation of the rat heart and centrifuged at 10,000 g for 10 minutes at 4°C. Then 5 mL of plasma were decanted and used for infusion for all cardiac perfusion groups [19]. 

### 2.6. Isolation of PMNs

 PMNs were prepared from Male Sprague Dawley rats (350–400 g, Ace Animals, Boyertown, PA). After injection (i.p.) of 0.5% glycogen (Sigma Chemical Co., St. Louis, MO) for 16–18 hours, PMNs were harvested by peritoneal lavage in 30 mL of 0.9% NaCl and centrifuged to remove the debris, as previously described [17]. The PMN preparations were >90% pure and >95% viable, according to microscopic analysis and exclusion of 0.3% trypan blue, respectively.

### 2.7. Determination of PMN Vascular Adherence and Infiltration into the Cardiac Tissue

Three rat hearts that were closest to the group mean for the cardiac function studies from each group were used for histological analysis. The hearts were dehydrated in graded ice-cold acetone washes (50–100%) and embedded in plastic and sectioned into 2.5 *μ*m serial sections. Then sections were stained with hematoxylin and eosin by previously established methods [17]. Under light microscopy, 10 areas of each rat heart from the left ventricle were counted for PMN vascular adherence and infiltration into the heart tissue and expressed as adhered and total PMNs/mm^2^. 

### 2.8. Statistical Analysis

All data in the text and figures were presented as means ± SEM. The data from more than two groups were analyzed by ANOVA. The data from only two groups were analyzed by student *t*-test. Probability values of <.05 were considered to be statistically significant.

## 3. Results

### 3.1. BH_4_ or BH_2_ on Endothelial NO Release

NO release from rat nonischemic aortic endothelium was measured to determine the effects of BH_4_ or BH_2_ on NO release. Moreover, the dose-responses of BH_4_ and BH_2_ on NO release also provide the optimal dose ranges for testing their effects in oxidative stress and postreperfused cardiac function during I/R. As shown in Table 1, BH_4_ dose dependently increased NO release, whereas BH_2_ dose dependently decreased NO release from aortic segments. The basal endothelial NO release was 2.28 ± 0.25 picomoles NO/mg tissue. BH_4_ (10 *μ*M, 20 *μ*M, and 40 *μ*M) produced a significant increase in NO release above basal to 4.45 ± 0.43 (*P* < .05), 4.88 ± 0.50 (*P* < .01), and 5.01 ± 0.61 (*P* < .01) picomoles NO/mg tissue, respectively. By contrast, BH_2_ (50 *μ*M, 100 *μ*M and 200 *μ*M) dose dependently decreased endothelial NO release. The decreases in NO release by BH_2_ were significant from the increasing NO releases by BH_4_ (all *P* < .01). Ach (5 *μ*M) was used as a positive control in this assay and stimulated the endothelium causing an increase to 5.38 ± 0.70 picomoles NO/mg tissue (*P* < .01). The NOS inhibitor, L-NAME (800 *μ*M), served as negative control, and significantly blocked NO release from aortic endothelium in presence of Ach and BH_4_.

### 3.2. Effects of BH_4_ or BH_2_ on H_2_O_2_ Release from Rat Femoral Veins during Reperfusion

**Figure 2:**
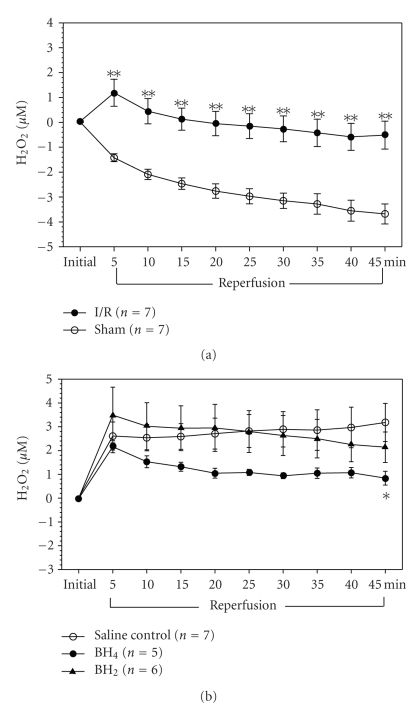
Measurement of H_2_O_2_ (*μ*M) release from rat femoral veins during reperfusion in the saline control group and BH_4_- or BH_2_-treated group. (a) H_2_O_2_ (*μ*M) releases from rat sham and I/R femoral veins during reperfusion in the saline control group. Anesthetized rats were given saline via tail vein at the beginning of reperfusion (*n* = 7). There was a significant increase in H_2_O_2_ release from I/R veins compared to sham veins during reperfusion. (**P < .01 from sham limb) (b) The relative difference in H_2_O_2_ release between I/R and sham femoral veins in the saline control group (*n* = 7), BH_4_ (6.5 mg/kg) treated group (*n* = 5), and BH_2_ (2 mg/kg) treated group (*n* = 6). BH_4_ or BH_2_ was given via tail vein at the beginning of reperfusion. BH_4_ treatment significantly decreased H_2_O_2_ release at 45-minute reperfusion compared to saline control. (*P < .05 from saline control group).

 The femoral vein subjected to I/R in saline control rats exhibited a significant increase in H_2_O_2_ release (i.e., up to 3.2 *μ*M) throughout reperfusion compared to sham vein (Figure 2(a)). This data supports the concept that oxidative stress is increased during reperfusion. Furthermore, H_2_O_2_ release increased in 2 mg/kg BH_2_-treated animals similar to saline control (Figure 2(b)) in that H_2_O_2_ release significantly increased in the I/R limb relative to the sham limb throughout the 45 minutes of reperfusion. H_2_O_2_ release increased in the saline and BH_2_-treated animals by 2.6 *μ*M and 3.5 *μ*M, respectively during the first 5 minutes of reperfusion and remained elevated by 3.2 *μ*M and 2.1 *μ*M, respectively, by 45 minutes of reperfusion (Figure 2(b)). By contrast, applying 6.5 mg/kg BH_4_ at the start of reperfusion decreased H_2_O_2_ release (i.e., down to 0.84 *μ*M) in the femoral I/R vein during the whole 45 minutes of reperfusion which was significant at 45 minutes of reperfusion in comparison with the saline control femoral I/R vein regarding the relative difference in each of their sham femoral veins, respectively (Figure 2(b)). These findings indicated that BH_4_ applied at the beginning of reperfusion decreased the oxidative stress during reperfusion, whereas BH_2_ administration was similar to saline control and did not further increase the oxidative stress.

### 3.3. Effects of BH_4_ or BH_2_ on NO Release from Rat Femoral Veins during Reperfusion

**Figure 3:**
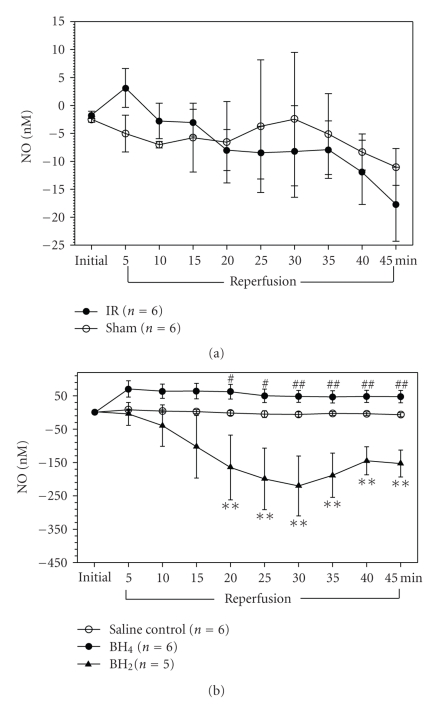
Measurement of NO (nM) release from rat femoral veins during reperfusion in the saline control group and BH_4_- or BH_2_-treated group. (a) NO (nM) releases from rat sham and I/R femoral veins during reperfusion in the saline control group. Anesthetized rats were given saline via tail vein at the beginning of reperfusion (*n* = 6). There was a slight decrease in NO release from I/R veins compared to sham veins after 20 minutes of reperfusion. (b) The relative difference in NO release between I/R and sham femoral veins in the saline control group (*n* = 6), BH_4_ (6.5 mg/kg)-treated group (*n* = 6), and BH_2_ (4 mg/kg)-treated group (*n* = 5). BH_4_ or BH_2_ was given via tail vein at the beginning of reperfusion. BH_2_ treatment significantly decreased NO release from 20 minutes and throughout the rest of reperfusion. BH_4_ treatment significantly increased NO release from 20 minutes to 45-minute reperfusion compared to BH_2_ treatment. (*P < .05, **P < .01 from saline control group; ^#^ *P* < .05, ^##^ *P* < .01 from BH_2_-treated group).

Compared to sham vein, the femoral vein subjected to I/R in saline control rats exhibited a slight increase in NO release for the first 5 minutes of reperfusion (i.e., up to 8.2 nM), and then gradually decreased for the rest of reperfusion (i.e., decreased by 6.6 nM from baseline) (Figure 3(a)). The initial increase may be due to the shear wall stress after restoration of blood flow. Applying 4 mg/kg BH_2_ at the beginning of reperfusion significantly decreased NO release (i.e., decreased by 220 nM) from 20 minutes to 45 minutes of reperfusion (all P < .01) in comparison with the saline control femoral I/R vein regarding the relative difference in each of their sham femoral veins, respectively (Figure 3(b)). By contrast, 6.5 mg/kg BH_4_ showed a significant increase in NO release from 20 minutes (*P* < .05) to 45 minutes reperfusion (*P* < .01) (i.e., up to 268 nM) comparing to BH_2_ treatment (Figure 3(b)). This data indicated that BH2 applied at the beginning of reperfusion significantly decreased NO release during reperfusion, whereas BH_4_ administration significantly increased NO release compared to BH_2_ treatment.

### 3.4. Cardiac Effects of BH_4_ or BH_2_ on PMN-Induced MI/R Injury

**Figure 4:**
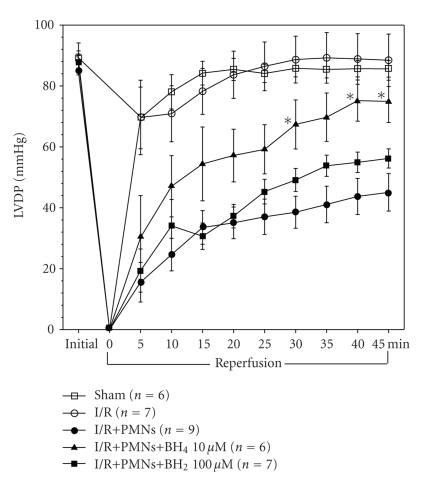
Time course of cardiac function parameters (LVDP) from sham, I/R, I/R+PMN, I/R+PMN+BH_4_ (10 *μ*M), and I/R+PMN+BH_2_ (100 *μ*M) groups. There was no significant difference in baseline values among these groups. The sham group (*n* = 6) maintained the same LVDP throughout the 80 minutes protocol. The I/R+PMN (*n* = 9) and I/R+PMN+BH_2_  (*n* = 7) groups exhibited a significant and sustained reduction in LVDP during the 45 minutes of reperfusion compared to sham. I/R (*n* = 7) and I/R+PMN+BH_4_  (*n* = 6) groups exhibited significant restoration of LVDP during the 45 minutes reperfusion compared to I/R+PMN group. All values are expressed as mean ± SEM. (*P < .05, from I/R+PMN values).

Different doses of BH_4_ or BH_2_, were applied individually to the isolated PMN-induced MI/R hearts to determine their effects on postreperfused cardiac function. Figure 4 shows the time course of cardiac contractile function (LVDP) for the sham, I/R, I/R+PMN, I/R+PMN+BH_4_ (10 *μ*M), and I/R+PMN+BH_2_ (100 *μ*M) groups. It illustrates the changes in LVDP during the 80-minute perfusion period. The hearts in the sham group maintained LVDP throughout the entire duration of the perfusion period (96 ± 3% of initial baseline values). I/R group hearts experienced a minor depression in LVDP (i.e., down to 79% of initial baseline) during the initial 5 minutes of reperfusion, but by the end of reperfusion they had recovered to 100 ± 10% of initial baseline values. By contrast, hearts in the I/R+PMN group exhibited sustained cardiac contractile dysfunction, only recovering to 53 ± 7% of initial baseline values at 45-minute reperfusion. However, BH_4_ (10 *μ*M) given during early reperfusion (i.e., first 5 minutes) significantly increased LVDP at 30, 40, and 45 minutes postreperfusion compared to I/R+PMN hearts (all *P* < .05) and recovered to 85 ± 8% of initial baseline. By contrast, LVDP time course in BH_2_ (100 *μ*M)-treated group was similar to that of I/R+PMN group (Figure 4). This data suggests that BH_4_ treatment, not BH_2_ treatment, provided significant restoration in postreperfused LVDP. 

**Figure 5:**
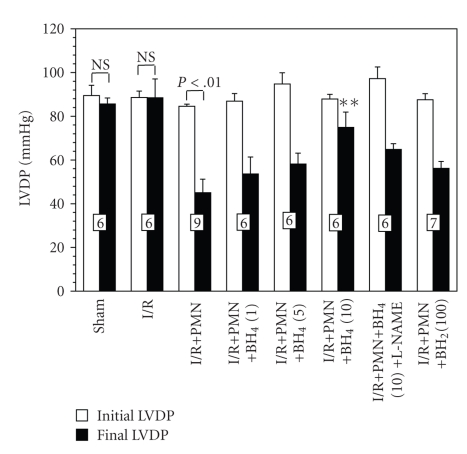
Initial and final LVDP expressed in mmHg from different experimental groups. Hearts were perfused in the presence or absence of PMNs. PMNs induced a significant decrease in LVDP, which was attenuated by the BH_4_ (10 *μ*M), not by BH_2_ (100 *μ*M). L-NAME (50 *μ*M) blocked the cardioprotective effect of BH_4_ treatment. All values are expressed as mean ± SEM. Numbers of hearts are at the bottom of the bars. All drugs are in *μ*M concentrations. (**P < .01 from final I/R+PMN values; NS, not significant compared to initial LVDP values).

**Figure 6:**
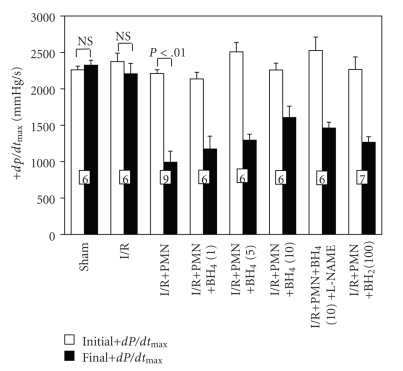
Initial and final +*dP*/*dt*_max_ expressed in mmHg/s from different experimental groups. Hearts were perfused in the presence or absence of PMNs. PMNs induced a significant decrease in +*dP*/*dt*_max_. Neither BH_4_ (10 *μ*M) nor BH_2_ (100 *μ*M) treatment significantly improved +*dP*/*dt*_max_ compared to I/R+PMN hearts. All values are expressed as mean ± SEM. Numbers of hearts are at the bottom of the bars. All drugs are in *μ*M concentrations. (NS, not significant compared to initial LVDP values).

Figures 5 and 6 showed the initial and final values for LVDP and +*dP*/*dt*_max_ from the control and different treated groups, respectively. There was no significant difference between the initial baseline values of all the groups studied. There was also no significant difference between the initial and final values of LVDP and +*dP*/*dt*_max_ for the sham and I/R groups. In order to establish whether the BH_4_ or BH_2_ produced any direct inotropic effects on cardiac contractile function, sham and I/R hearts were perfused with BH_4_ (10 *μ*M) or BH_2_ (100 *μ*M). The BH_4_ (10 *μ*M) or BH_2_ (100 *μ*M) treated sham and I/R hearts maintained a similar cardiac function with respect to initial and final LVDP and +*dP*/*dt*_max_, indicating that BH_4_ (10 *μ*M) or BH_2_ (100 *μ*M) has no direct effect on cardiac contractile function, even in the setting of I/R without PMNs (data not shown).

 However, I/R+PMN hearts only recovered to 53 ± 7% of initial LVDP and 45 ± 7% of initial +*dP*/*dt*_max_ at 45 minutes post-reperfusion, both were significantly lower than initial baselines (Figures 5 and 6, both *P* < .01). This result suggests that PMNs are principally responsible for the sustained cardiac contractile dysfunction in this model of MI/R. We have previously shown that the amount of PMNs used in this model does not elicit cardiodepressant effects in sham hearts [19]. Collectively, these data indicate that the combination of 20-minute ischemia followed by 45-minute reperfusion in the presence of PMNs is required to sustain cardiac contractile dysfunction.

 To test the effect of BH_4_ on the PMN-induced post I/R cardiac dysfunction, we applied this drug during the first 5 minutes of reperfusion. BH_4_ exerted a dose-dependent effect on restoring postreperfused LVDP and +*dP*/*dt*_max_. 1 and 5 *μ*M BH_4_ treated I/R+PMN hearts finally recovered to 62 ± 9% and 61 ± 5% of initial LVDP, respectively, and both were not significantly different from the final values of the I/R+PMN group (Figure 5). However, 10 *μ*M BH_4_ exhibited significant restoration in final LVDP (85 ± 8% of initial baseline) compared to that of I/R+PMN hearts (53 ± 7% of initial baseline). By contrast, final +*dP*/*dt*_max_ in 1, 5, and 10 *μ*M BH_4_-treated I/R+PMN hearts were 55 ± 8%, 52 ± 3%, and 71 ± 7% of initial values, respectively, but were not significantly different from the final +*dP*/*dt*_max_ of the I/R+PMN hearts (Figure 6). This data suggests that BH_4_ treatment during the early reperfusion can partially improve post I/R cardiac contractile function. 

 On the other hand, application of BH_2_ (100 *μ*M) during the first 5 minutes of reperfusion did not restore postreperfusion cardiac contractile function in the presence of activated PMNs. The I/R+PMN+BH_2_ (100 *μ*M) hearts recovered to 64 ± 4% and 56 ± 3% for LVDP and +*dP*/*dt*_max_ of initial baseline at 45-minute postreperfusion, respectively. These values were not significantly different from the final values of I/R+PMN control group (Figures 5 and 6). Moreover, the cardiac function in BH_2_-treated animals was not different from I/R+PMN control hearts at any time point during the reperfusion. This data suggests that BH_2_ treatment exhibited the similar compromised postreperfused cardiac function as the I/R+PMN control group.

### 3.5. Effects of L-NAME on Cardioprotective Effects of BH_4_

To further determine if a NO mechanism is involved in the cardioprotection of BH_4_ (10 *μ*M) application during reperfusion, a NOS inhibitor, L-NAME (50 *μ*M), was perfused throughout the 45-minute reperfusion. Previous studies have shown that L-NAME given by itself during reperfusion is not associated with cardioprotection [23]. The cardioprotective effects of BH_4_ (10 *μ*M) were blocked by L-NAME suggesting that a NO mechanism was principally responsible for mediating the cardioprotective effects of BH_4_. The final LVDP and +*dP*/*dt*_max_ values in this group of hearts were only 67 ± 3% and 58 ± 3% of the initial baseline, respectively, and were not significantly different from the final values of the IR+PMN group (Figures 5 and 6).

### 3.6. Effects of BH_4_ or BH_2_ on Postreperfused PMNs Adherence/Infiltration

**Figure 7:**
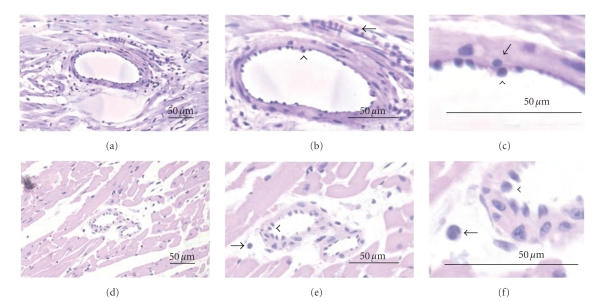
Light microscopy photograph of hematoxylin and eosin-stained rat heart tissue from I/R+PMN and I/R+PMN+BH_4_ (10 *μ*M). (a)–(c), I/R+PMN heart illustrated enhanced PMN coronary vascular adherence and tissue infiltration ((a): 20x; (b): 40x; (c): 100x). (d)–(f), I/R+PMN+BH_4_ (10 *μ*M) heart exhibited a reduction in PMN coronary vascular adherence and tissue infiltration compared with I/R+PMN ((d): 20x; (e): 40x; (f): 100x). Arrows indicate infiltrated PMN and arrowheads indicate PMN adherence to coronary vasculature. Scale bar, 50 *μ*m.

 The infiltration of PMNs into the myocardium within the 45-minute reperfusion period is closely correlated with the cardiac injury associated with I/R in this model. Therefore, PMN vascular adherence and myocardial infiltration were determined for each experimental group. The representative PMN coronary vascular adherence and transmigration from I/R+PMN and I/R+PMN+ BH_4_ (10 *μ*M) groups were shown by light microscopy under 20x, 40x, and 100x magnification (Figure 7). Arrowheads indicate PMN adherence to the coronary vascular endothelium, while arrows indicate PMNs that have infiltrated into the myocardium. I/R+PMN+BH_4_ (10 *μ*M) hearts displayed a considerable reduction in PMN vascular adherence and tissue infiltration compared to I/R+PMN hearts.

**Figure 8:**
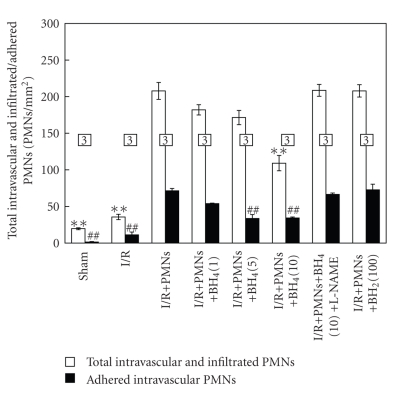
Histological assessment of total intravascular/infiltrated PMNs and vascular adhered PMNs in isolated postreperfused rat heart tissue from different experimental groups. The total intravascular/infiltrated PMNs in sham, I/R and I/R+PMN+BH_4_ (10 *μ*M) groups were significantly reduced compared to that in I/R+PMN group. Similarly, the vascular adhered PMNs in sham, I/R, I/R+PMN+BH_4_ (5 *μ*M), and I/R+PMN+BH_4_ (10 *μ*M) groups were also significantly reduced compared to that in I/R+PMN group. All values are expressed as mean ± SEM. Numbers of samples are listed in the box with the bars. All drugs are in *μ*M concentrations. (**P < .01 from total intravascular/infiltrated PMNs in I/R+PMN group; ^##^ *P* < .01 from vascular adhered PMNs in I/R+PMN group).

The total and adhered intravascular PMNs from different experimental groups are shown in Figure 8. Sham and I/R hearts exhibited very few vascular adhered and transmigrated PMNs in the postreperfused heart tissue, which represents only resident PMNs. However, I/R+PMN hearts exhibited a significantly increased total intravascular and infiltrated PMNs (208 PMNs/mm^2^ in I/R+PMN versus 20 PMNs/mm^2^ in sham), as well as vascular adhered PMNs (71 PMNs/mm^2^ in I/R+PMN versus <2 PMNs/mm^2^ in sham). Similarly, BH_2_-treated hearts also showed significantly higher total (208 PMNs/mm^2^) and vascular adhered PMNs (73 PMNs/mm^2^). By contrast, BH_4_ treated hearts exhibited a dose-dependent decrease in total and vascular adhered PMNs. The I/R+PMN+BH_4_ (10 *μ*M) hearts showed significantly less adhered PMNs (34 PMNs/mm^2^) and total PMNs (109 PMNs/mm^2^) compared to those of I/R+PMN hearts (both *P* < .01). Furthermore, L-NAME blocked the attenuation of total intravascular and infiltrated PMNs by BH_4_ application during reperfusion (Figure 8). 

## 4. Discussion

### 4.1. Summary of Major Findings

 The major findings of this study were as follows. (1) BH_4_ promoted NO release from vascular endothelium in a dose-dependent manner, whereas BH_2_ decreased NO release in nonischemic rat aortic segments. (2) The increased oxidative stress (i.e., H_2_O_2_) during reperfusion was significantly decreased by BH_4_ (6.5 mg/kg) in femoral I/R veins in vivo. (3) BH_2_ (4 mg/kg) applied at the beginning of reperfusion significantly decreased NO release during reperfusion, whereas BH_4_ (6.5 mg/kg) significantly increased NO release compared to BH_2_ treatment in femoral I/R veins in vivo. (4) BH_4_ (10 *μ*M), not BH_2_ (100 *μ*M) treated I/R+PMN hearts, exerted significant restoration in LVDP, which was associated with significant attenuation of intravascular PMN adherence/infiltration in postreperfused myocardium. (5) The cardioprotective effects of BH_4_ on cardiac contractile function and infiltrated/adhered PMNs were blocked by L-NAME.

### 4.2. Contribution of BH_4_ to eNOS Coupling and BH_2_ to eNOS Uncoupling

**Figure 9:**
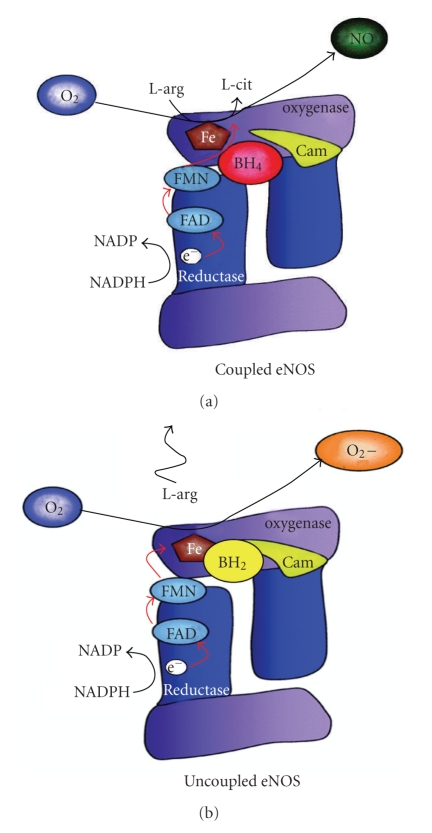
Schematic diagram of eNOS coupling and uncoupling. BH_4_ binds to the heme oxygenase domain to promote L-arginine substrate binding under eNOS coupling conditions. By contrast, BH_2_ competes with BH_4_ at the heme oxygenase domain and promotes molecular oxygen as substrate to produce superoxide and eNOS uncoupling conditions (Adopted from [9]).

It is well known that BH_4_ is an essential cofactor of eNOS to produce NO by maintaining eNOS in the coupled state via facilitating the binding of L-arginine to the heme site of eNOS [7, 9]. By contrast, BH_2_, or decreased ratio between BH_4_ and BH_2_, can result in the uncoupled state of eNOS in that L-arginine is not bound to the heme site and molecular oxygen is used as the substrate to produce SO instead of NO (see Figure 9) [9–11, 13]. Therefore, in the nonischemic aortic segments, we found that BH_4_ dose dependently increased NO release by promoting eNOS coupling to produce more NO. By contrast, BH_2_ dose dependently decreased NO release from aortic segments. This trend of decreasing NO bioavailability by BH_2_ may be because BH_2_ promotes eNOS uncoupling and facilitates eNOS to produce SO instead of NO, which further quenches NO via the formation of peroxynitrite [9]. 

Furthermore, we found similar effects of BH_4_ and BH_2_ on NO release in femoral I/R. There was a slight decrease in NO release (i.e., 6-7 nM) from femoral veins in the I/R limb compared to the sham limb in the saline control group. By contrast, applying BH_2_ at the beginning of reperfusion significantly decreased NO release from 15 minutes and throughout the rest of reperfusion. However, administration of BH_4_ during the early reperfusion significantly increased NO release in comparison to BH_2_ treatment. This data suggests that under I/R conditions, BH_2_ can further facilitate eNOS uncoupling and further reduce NO release (i.e., in nM concentration), whereas BH_4_ may help eNOS maintain the coupled state and increase NO bioavailability. The in vivo data from this study further supports in vitro data regarding the role of BH_4_ and BH_2_ in eNOS coupling and uncoupling [7, 11].

On the other hand, the effects of BH_4_ and BH_2_ on H_2_O_2_ release in femoral I/R also demonstrate the contribution of eNOS to the oxidative stress during reperfusion. It is well known that H_2_O_2_ is a good indicator of oxidative stress with a longer half-life (i.e., min) compared to SO (i.e., sec) [12]. By directly measuring H_2_O_2_ release from femoral veins in a rat femoral I/R model, we found that H_2_O_2_ release from the I/R limb was significantly higher throughout the whole 45-minute reperfusion period compared to the sham limb. This result provides direct evidence to show the oxidative stress during the early reperfusion. Furthermore, the administration of BH_4_ at the beginning of reperfusion significantly decreased H_2_O_2_ release. This data suggests that maintenance of eNOS coupling by BH_4_ at the start of reperfusion would help to attenuate the oxidative stress. 

 By contrast, BH_2_ treatment during reperfusion maintained eNOS uncoupling, which is involved in mediating oxidative stress observed during the reperfusion. BH_2_ treatment resulted in the similar H_2_O_2_ increase to the saline control throughout the reperfusion. It is possible that increasing BH_2_ to BH_4_ ratio by itself may not make a significant contribution to enhance the oxidative stress release compared to the saline control. As shown previously, BH_2_ significantly decreased NO release compared to saline control during reperfusion, and this decrease was in the range of 150 to 200 nM, which is 10 to 15 times less the amount of H_2_O_2_ change (i.e., micromolar range). It may be plausible to speculate that significant changes in NO release in the nanomolar range can be observed with BH_2_ compared to micromolar differences in H_2_O_2_ release. It is well known that BH_2_ binds with equal affinity as BH_4_ to the eNOS oxygenase domain, and can displace BH_4 _binding to facilitate eNOS uncoupling [13]. To further promote additional eNOS uncoupling, it would be necessary to increase eNOS activity, such as with a protein kinase C (PKC) epsilon activator, combined with additional BH_2_. Conversely, supplementing BH_4_ under increased eNOS activity conditions may promote additional eNOS coupling that otherwise would not be observed under constitutive eNOS activity conditions [24]. These experiments would be important to identify the overall significance of eNOS coupling/uncoupling contribution to the restoration/impairment of blood vessel and organ function during reperfusion. Moreover, these experiments would also identify a potential therapeutic tool that could be used clinically in patients suffering from reperfusion injury by ischemic-induced heart attack, coronary bypass/angioplasty and organ transplantation.

### 4.3. The Role of BH_4_ and BH_2_ on Postreperfused Cardiac Function and PMN Adherence/Infiltration

Previous studies have shown the effectiveness of BH_4_ in restoring postreperfused cardiac function [15]. However, these studies also gave BH_4_ prior to ischemia in addition to giving BH_4_ during the reperfusion. By contrast, our studies only gave BH_4_ during reperfusion. This point is important in that pretreatment of tissue or organs prior to invasive surgical procedures (i.e., organ transplantation) is not always a feasible option. Therefore, in our study, BH_4_ is only given during the first 5 minutes of reperfusion. We found that BH_4_ treatment exerted partial restoration in postreperfused cardiac function, which is associated with significantly less PMN vascular adherence and tissue infiltration. The uniqueness of our study is that we only evaluate the effects of BH_4_ during the reperfusion phase and clinically this approach may be more feasible in that BH_4_ could be administered immediately following coronary artery bypass and organ transplantation procedures. 

Despite that BH_4_-treated I/R+PMN hearts exhibited 85% postreperfused LVDP, +*dP*/*dt*_max_ only recovered by 71% and was not significantly increased compared to I/R+PMN control hearts. This may be due to saturation of eNOS coupling with adding BH_4_ which may slow down the recovery of postreperfused +*dP*/*dt*_max_. By contrast, BH_2_-treated hearts did not show any improvements in postreperfused heart function suggesting that BH_2_ maintained eNOS uncoupling. The postreperfused heart function in BH_2_-treated hearts was similar to I/R+PMN control hearts, which suggest that BH_2_ did not further increase oxidative stress and is consistent with the femoral I/R H_2_O_2_ release data. In future studies, we plan to evaluate the role of eNOS uncoupling by increasing eNOS activity combined with BH_2_ for any potential additive or synergistic effect. To test this hypothesis, increasing eNOS activity during reperfusion with a PKC epsilon activator combined with the same concentration of BH_2_ should result in greater oxidative stress and lead to a more compromised postreperfused cardiac function since these conditions would promote more BH_2_ to interact with eNOS [25]. Conversely, adding BH_4_ with increased eNOS activity (i.e., PKC epsilon activator) would promote more BH_4_ to interact with eNOS to produce more NO and optimally improve postreperfused +*dP*/*dt*_max_.

Alternatively, inhibiting eNOS only during reperfusion with a PKC epsilon inhibitor would also attenuate oxidative stress and restore postreperfused cardiac function regardless of BH_4_-to-BH_2_ ratio as suggested from previous work showing restoration of postreperfused cardiac function with PKC epsilon inhibitor in an acute I/R and heart transplantation models [12, 26]. In contrast to PKC epsilon inhibitor, L-NAME does not inhibit SO release from eNOS [11]. L-NAME competes with L-arginine at the heme site of the eNOS oxygenase domain to inhibit the production of NO. However, L-NAME will not inhibit the electron reduction of molecular oxygen to SO at the site when BH_2_-to-BH_4_ ratio is increased [11]. It is conceivable that L-NAME would therefore block the cardioprotective effects of BH_4_ since it is competing with L-arginine as a substrate as shown in Figures 5 and 6.

### 4.4. Significance of Findings

In summary, this study further supports the conception of BH_4_ promoting eNOS coupling during I/R. By contrast, BH_2_ treatment may maintain the eNOS uncoupled state during I/R and is associated with compromised postreperfused cardiac function and increased oxidative stress. Moreover, the connection of eNOS coupling or uncoupling to I/R injury, especially during the reperfusion, may encourage a novel optimal treatment strategy for clinical cases.

## Figures and Tables

**Table 1:** The effects of BH_4_ or BH_2_ on NO release from nonischemic rat aortic segments.

Basal/Treatments	NO release (picomoles/mg tissue)	Sample No.
Basal	2.28 ± 0.25	*n* = 17
Ach (5 *μ*M)	5.38 ± 0.70**	*n* = 20
BH_4_ (1 *μ*M)	2.74 ± 0.16	*n* = 14
BH_4_ (10 *μ*M)	4.45 ± 0.43*	*n* = 21
BH_4_ (20 *μ*M)	4.88 ± 0.50**	*n* = 24
BH_4_ (40 *μ*M)	5.01 ± 0.61**	*n* = 21
BH_2_ (50 *μ*M)	0.87 ± 0.33^##^	*n* = 21
BH_2_ (100 *μ*M)	0.86 ± 0.23^##^	*n* = 25
BH_2_ (200 *μ*M)	0.78 ± 0.33^##^	*n* = 14
Ach (5 *μ*M)+L-NAME (800 *μ*M)	−0.59 ± 0.38	*n* = 5
BH_4_ (10 *μ*M)+L-NAME (800 *μ*M)	−1.14 ± 0.48	*n* = 8
BH_2_ (100 *μ*M)+L-NAME (800 *μ*M)	−1.70 ± 0.34	*n* = 8

*P < .05, **P < .01 compared to basal NO release; ^##^ *P* < .01 compared to the effects of BH_4_ on NO release.

**Table 2:** Experimental groups in observation of postreperfused cardiac function from the isolated perfused rat heart.

Group	Sham I/R	I/R	I/R+PMN	I/R+PMN+L-NAME
Control (No Drug)	*n* = 6	*n* = 7	*n* = 9	
BH_4_ (1 *μ*M)			*n* = 6	
BH_4_ (5 *μ*M)			*n* = 6	
BH_4_ (10 *μ*M)	*n* = 6	*n* = 7	*n* = 6	*n* = 6
BH_2_ (100 *μ*M)	*n* = 4	*n* = 5	*n* = 7	
